# Conserved residues of the immunosuppressive domain of MLV are essential for regulating the fusion-critical SU-TM disulfide bond

**DOI:** 10.1128/jvi.00989-24

**Published:** 2024-10-29

**Authors:** Victoria A. Hogan, Julia Harmon, Miguel Cid-Rosas, Laura R. Hall, Welkin E. Johnson

**Affiliations:** 1 Department of Biology, Boston College, Chestnut Hill, Massachusetts, USA; 2 Dana-Farber Cancer Institute, Boston, Massachusetts, USA; 3 Chan Zuckerberg Biohub, San Francisco, California, USA; The Ohio State University, Columbus, Ohio, USA

**Keywords:** envelope glycoprotein, immunosuppressive domain, murine leukemia virus, fusion, infectivity, gamma-type Env

## Abstract

The Env protein of murine leukemia virus (MLV) is the prototype of a large clade of retroviral fusogens, collectively known as gamma-type Envs. Gamma-type Envs are found in retroviruses and endogenous retroviruses (ERVs) representing a broad range of vertebrate hosts. All gamma-type Envs contain a highly conserved stretch of 26-residues in the transmembrane subunit (TM) comprising two motifs, a putative immunosuppressive domain (ISD) and a CX_6_CC motif. Extraordinary conservation of the ISD and its invariant association with the CX_6_CC suggests a fundamental contribution to Env function. To investigate ISD function, we characterized several mutants with single amino acid substitutions at conserved positions in the MLV ISD. A majority abolished infectivity, although we did not observe a corresponding loss in intrinsic ability to mediate membrane fusion. Ratios of the surface subunit (SU) to capsid protein (CA) in virions were diminished for a majority of the ISD mutants, while TM:CA ratios were similar to wild type. Specific loss of SU reflected premature isomerization of the labile disulfide bond that links SU and TM prior to fusion. Indeed, all non-infectious mutants displayed significantly lower disulfide stability than wild-type Env. These results reveal a role for ISD positions 2, 3, 4, 7, and 10 in regulating a late step in entry after fusion peptide insertion but prior to creation of the fusion pore. This implies that the ISD is part of a larger domain, comprising the ISD and CX_6_CC motifs, that is critical for the formation and regulation of the metastable, intersubunit disulfide bond.

**IMPORTANCE:**

The gamma-type Env is a prevalent viral fusogen, found within retroviruses and endogenous retroviruses across vertebrate species and in filoviruses such as Ebolavirus. The fusion mechanism of gamma-type Envs is unique from other Class I fusogens such as those of influenza A virus and HIV-1. Gamma-type Envs contain a hallmark feature known as the immunosuppressive domain (ISD) that has been the subject of some controversy in the literature surrounding its putative immunosuppressive effects. Despite the distinctive conservation of the ISD, little has been done to investigate the role of this region for the function of this widespread fusogen. Our work demonstrates the importance of the ISD for the function of gamma-type Envs in infection, particularly in regulating the intermediate steps of membrane fusion. Understanding the fusion mechanism of gamma-type Envs has broad implications for understanding the entry of extant viruses and aspects of host biology connected to co-opted endogenous gamma-type Envs.

## INTRODUCTION

Retroviruses use Envelope (Env) glycoproteins embedded into the viral membrane to recognize host cell receptors and mediate membrane fusion. The fusion of the viral membrane with the host cell membrane allows entry of the viral capsid core into the host cell (1). Retroviral Envs are class I fusogens. The prototypical class I fusogens include HA of influenza A virus (IAV) and the envelope glycoprotein (Env) of HIV-1 (2). However, these paradigms lack features associated with a large subset of Class I fusion proteins that use a distinct mechanism for fusion and entry (3–5). These are the gamma-type Env fusion proteins. Gamma-type Envs are typified by Gammaretroviruses for which they are named but are also found in retroviruses representing *Alpharetrovirus*, *Betaretrovirus*, and *Deltaretrovirus* genera. Gammaretroviruses have been found to infect fish, amphibians, birds, and mammals, indicative of how the boundary between species has been, and likely remains, an easily traversable barrier for these viruses. Gamma-type Envs are further found across host species within endogenous loci [endogenous retroviruses (ERVs)] (6–8), and these endogenous gamma-type Envs have sometimes been co-opted during vertebrate evolution for a host function (9, 10). An example includes the human Syncytin proteins, which play a crucial role in placenta formation (11–14). In addition to retroviruses, gamma-type fusogens are also found in the family filoviridae, including the Ebolavirus (EBOV) GP (15).

**Fig 1:**
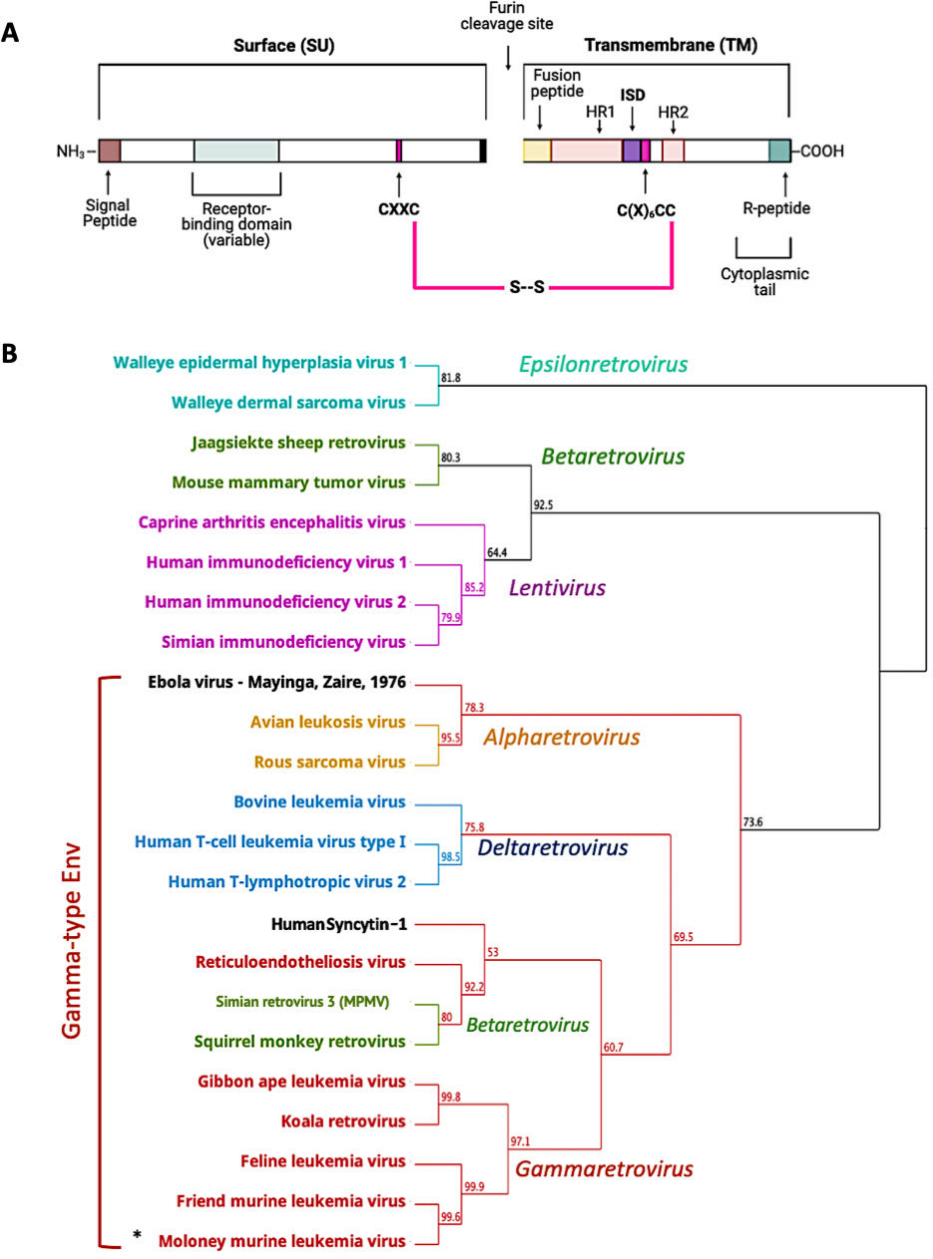
Gamma-type Envs are shared across multiple genera in the retroviridae and beyond and contain a distinctive disulfide bond between SU and TM. (**A**) Retroviral Env sequence showcasing common features of murine leukemia virus (MLV) and related gamma-type Envs. The location of the receptor binding domain is based on MLV; however, the location of this region can be highly variable and is not completely defined for many Envs. Of note are two cysteine motifs that are joined via a disulfide bond (shown in pink). HR1 and HR2 correspond to the heptad repeats. ISD corresponds to the immunosuppressive domain. (**B**) Geneious Prime (v2022.2.2) generated neighbor-joining tree of envelope proteins using an alignment of TM only, colored by genus except for ebolavirus and syncytin-1. Bootstrap values are shown at branch points. The asterisk corresponds to the gammaretrovirus used for the experiments in this study.

Like all Class I fusogens, gamma-type Envs are trimers composed of three heterodimers each with a surface (SU) and transmembrane (TM) subunit. However, the gamma-type Env clade is marked by several distinctive features that are not found in prototypical Class I fusogens such as HIV-1 Env or IAV HA. Specifically, the disulfide bond that joins the two subunits of Env is a metastable bond that must isomerize prior to fusion (3, 16, 17). This labile disulfide bond is unique to gamma-type Envs and forms between two highly conserved, cysteine-containing motifs (17, 18). Within SU, the CXXC (commonly CWLC) lends its second cysteine to the bond, while the first cysteine is reserved for isomerization during fusion (Fig. 1A) (16, 18). Within TM there is a CX_6_CC motif, of which the last cysteine contributes to the intersubunit disulfide bond with SU (18). The other two cysteines in this motif form their own disulfide bond that is thought to be structurally essential for TM. The labile disulfide bond that holds SU and TM together is essential for infectivity and fusion (16). During fusion, after receptor binding, the N-terminal fusion peptide of TM inserts into the host cell membrane. Isomerization of the intersubunit disulfide bond between SU and TM results in an internal bond within the CXXC motif of SU (16, 19–21). The uncoupling of SU and TM caused by isomerization is believed to allow TM to undergo a final conformational change (referred to as “hair-pinning”) that brings the cellular and viral membranes together to facilitate fusion (1, 22, 23).

Interestingly, the CX_6_CC motif is invariably part of a larger conserved stretch of 26 residues within TM that includes the 17 residue immunosuppressive domain (ISD). Conveniently, high-resolution pre-fusion structures of EBOV GP trimers contain this region of Gp2 (24). Previous work with a small synthetic peptide, homologous to this 17 amino acid stretch just upstream of the murine leukemia virus (MLV) CX_6_CC, demonstrated inhibition of lymphocytes and natural killer cells *in vitro* (25–31). Later, experiments using full Env proteins demonstrated immunosuppressive effects *in vivo* using a murine model of tumor suppression (14, 32–34). This work, combined with the conservation of this region across gamma-type Envs, led to the proposal that the ISD is responsible for a variety of phenomena including viral immune evasion, tumor immune evasion, and, in the case of syncytins, maternal-fetal tolerance.

Here, we report that the ISD and CX_6_CC motifs are always adjacent and invariably co-occur with the labile disulfide bond between SU and TM. Additionally, our results suggest that this 26-amino-acid region (spanning both the ISD and CX_6_CC) may have been conserved due to a fundamental role in the regulation and stabilization of the intersubunit disulfide bond between SU and TM, which is crucial in controlling an intermediate step of fusion unique to gamma-type Envs. We specifically demonstrate that small conservative changes within the ISD are sufficient to abolish infectivity, revealing the extreme intolerance for deviation within this region for proper Env function. Interestingly, these mutations display little to no impact on the biogenesis of Env and its innate cell-cell fusion capability. Instead, this loss of infectivity is correlated with premature isomerization or instability of the disulfide bond between SU and TM suggesting a role for the ISD in regulating stability of Gamma-type Env proteins. This is in line with previous literature reporting that larger deletions or double mutations in this region have drastic impacts on infectivity (35, 36).

## RESULTS

### Exceptional conservation of the 26 residues comprising the ISD and CX_6_CC

Phylogenetic analysis based on the TM subunit, including representatives from all Orthoretroviral genera, demonstrates that the Env proteins of alpharetroviruses, gammaretroviruses, deltaretroviruses, and some betaretroviruses constitute a gamma-type Env clade (Fig. 1B) (15). The GP2 subunit of filovirus glycoproteins, including EBOV GP2 also cluster within the clade of gamma-type entry proteins (Fig. 1B).

**Fig 2:**
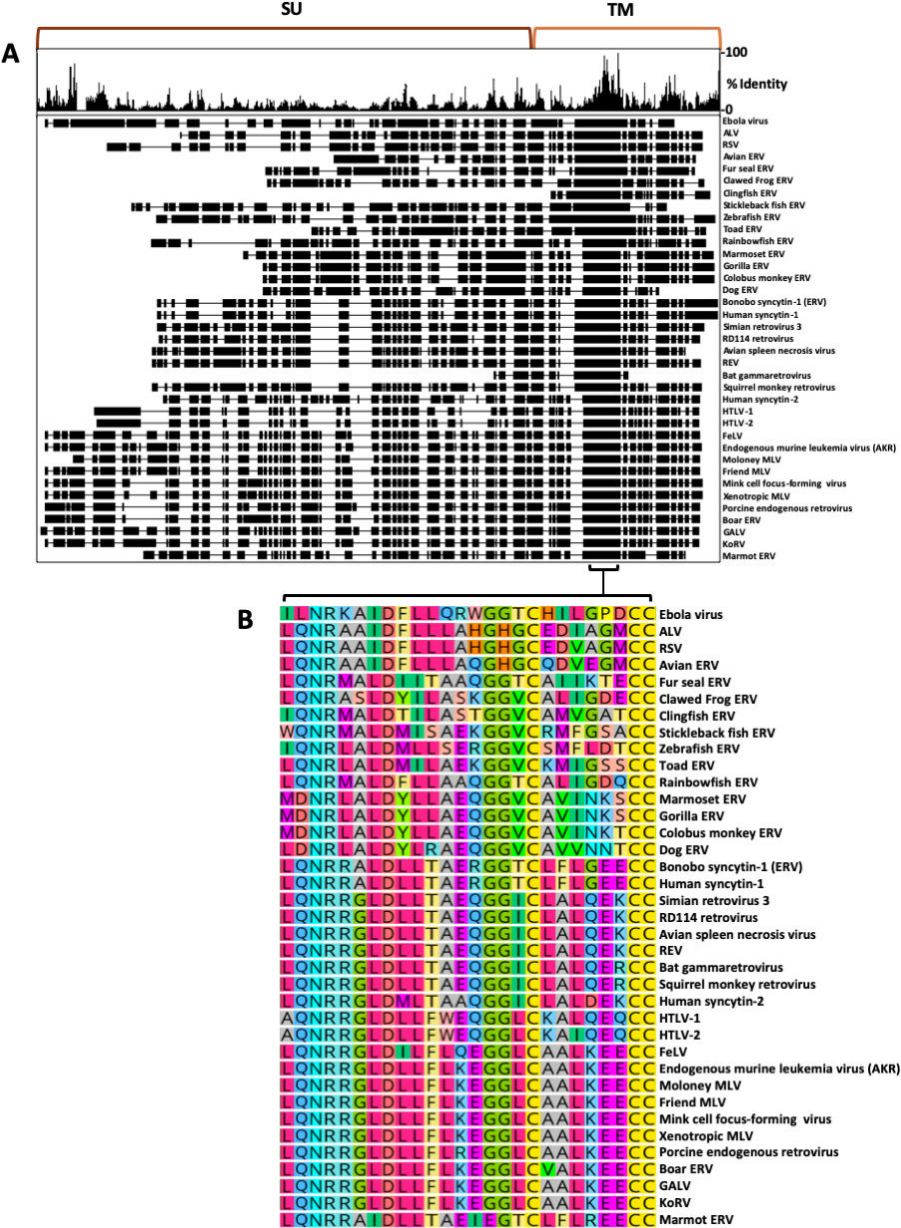
Conservation of the ISD and CX_6_CC motifs across highly divergent Env sequences. (**A**) Alignment of 37 whole or partial gamma-type entry protein sequences. Percent identity at each position is shown in the histogram (top). Black boxes indicate aligned residues, while horizontal lines indicate gaps in the alignment. Low overall identity is observed for SU and higher for TM. Alignment was generated using clustal omega as implemented in Geneious Prime (v2022.2.2). (**B**) The region with the highest conservation and longest unbroken span is shown zoomed in and contains the 26 amino acid stretch that comprises the ISD and CX_6_CC. The diversity of sequences used within the alignment represents hundreds of millions of years of evolution for both viruses and hosts.

Originally, the ISD was noted to be well conserved in a few frequently studied gamma-type Envs (25). To examine the conservation of the ISD in a broader evolutionary context, 36 protein sequences of gamma-type Envs and the EBOV GP, found in NCBI databases, were aligned using Clustal-Omega (Geneious Prime). Envs representing both exogenous viruses from multiple genera and Envs from ERV loci were included in the alignment. For the whole alignment, the average pairwise identity observed was 17%. In stark contrast to the rest of the Env alignment, a ~26 residue stretch within TM shows remarkable conservation (Fig. 2). This region corresponds to the ISD and the CX_6_CC motif, the latter of which is one of two cysteine-containing motifs within Env that form a critical disulfide bond between SU and TM. The length of the ISD and CX_6_CC together also remains constant, indicating that the two motifs were conserved together as part of a single 26 residue domain. This span of conservation holds the residues with the highest positional identity across the whole alignment and contains the only six residues with 100% pairwise identity—these correspond to MLV residues N540, R541, and D545 and the three cysteines of the CX_6_CC (C555, C562, and C563). Several other residues within the ISD have >75% pairwise identity, including ERV sequences.

For convenience, we number the positions of the ISD beginning with the leucine previously denoted as the start of the MLV ISD (25). Within the ISD, residues L1, Q2, N3, R4, L7, D8, L10, G15, and G16 show the highest conservation. The remaining positions are less well conserved yet still display strong similarity within phylogenetic clusters (Fig. 2B). These residues have not yet been resolved in a pre-fusion structure for MLV; however, pre-fusion structures of Ebola GP (6qd8) offer insight into their orientation, with positions 1, 2, 4, and 8 extending toward GP1 (SU) and positions 3, 7, and 10 projecting internally toward the central axis of the trimer (24).

Previous work demonstrated that certain mutations to the ISD and CX_6_CC (E14R and A20F) reduced infectivity connected with premature interactions with the receptor in the producing cell (14, 34, 36). This, combined with the ISD’s conservation with the disulfide forming CX_6_CC motif, prompted the hypothesis that the ISD may be conserved for an associated function in entry and disulfide bond stability/regulation.

### Individual mutations at each of the conserved sites of the ISD drastically reduce infectivity

**Fig 3:**
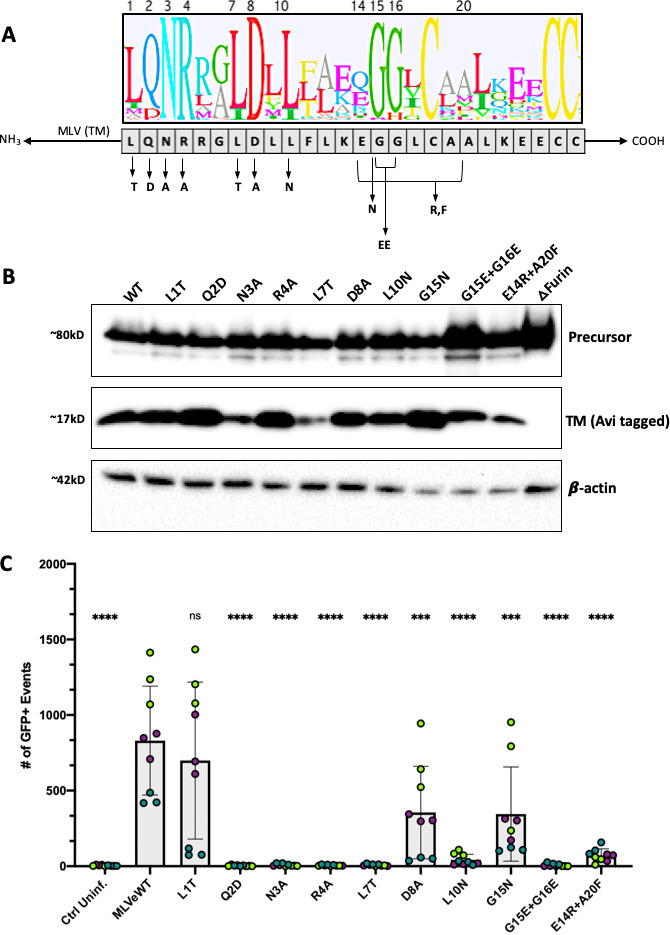
MLV ISD mutants are correctly processed but largely incapable of infection. (**A**) Mutagenic strategy to create eight single mutants and two double mutants to the most conserved regions. *Mutant E14R + A20F is not within the most conserved residues but instead replicates a mutant used previously in the literature (34, 36) believed to have no immunosuppression. Logo plot was based on alignment from Fig. 2 showing the conserved residues of the ISD. Leucine 1 of the ISD corresponds to L538 in Moloney MLV (accession NC_001501.1). (**B**) Expression of Env in whole cell lysates via reducing western blot of all ISD mutants. All Envs were C-terminally tagged with an Avi epitope tag for visualization. To specifically observe Env expression and processing, constructs encoding Env were transfected without Gag-Pol. Blots were stained with a rabbit anti-Avi polyclonal antibody to visualize TM and precursor. Env processing is demonstrated by the presence of cleaved TM at ~17 kD. A furin deletion mutant was used as a negative control. (**C**) Infectivity of each Envelope mutant pseudotyped with a Mo-MLV core and co-transfected with a packageable GFP plasmid. Virus was produced in 293T cells, and murine NIH-3T3 fibroblasts were used as target cells. GFP expression in target 3T3 cells, as measured by flow cytometry, was used as a measure of infection. Three infections, each performed in triplicate, are shown per condition. Data points are colored by infection round (**** =*P* < 0.0001, ns = not significant, ordinary one-way ANOVA with multiple comparisons).

To address the role of the ISD in Env function, several mutants were generated in the MLV Env by site-directed mutagenesis. Single amino acid changes were made to the most highly conserved residues within the ISD (Fig. 3A). All amino acid changes were made chosen to minimally impact protein structure, favoring polar to non-polar and charged to polar uncharged when possible. The mutant Q2D was chosen based by replacing the majority residue (glutamine) with one found in a minor subset of Envs (aspartate). An Avi epitope tag was added to the C-terminus of the Env coding sequences. Importantly, the complete TM sequence was retained including the R-peptide and protease cleavage site.

The glycine at position 15 was changed (G15N); however, as this residue is part of a double glycine, it was possible that the effects of a mutation here would be masked. Therefore, we additionally included a mutation eliminating both glycines (G15E + G16E).

Positions E14 and A20 are not highly conserved; however, the E14R + A20F mutant was previously reported as abrogating immunosuppression without compromising infectivity (14, 34), while another group reported a significant loss in infectivity (36). Because of this discrepancy, we included the E14R + A20F in our panel.

All mutant Envs were examined by transient transfection and visualization with western blot of whole cell lysates. A furin cleavage site deletion mutant was used as a negative control for processing. All mutant Envs were expressed and processed to a similar extent as revealed by the presence of a band correspond to the Env precursor polyprotein at ~80 kD and another band corresponding to the cleaved TM subunit at ~15 kD (Fig. 3B). As the SU subunit cannot be visualized with the Avi tag after processing, no SU was observed in Fig. 3B as expected.

Next, we tested the ability of the ISD mutants to facilitate infectivity of pseudotyped MLV virions in murine fibroblasts (NIH-3T3 cells). The majority of ISD mutant Envs were incapable of mediating infection. Except for mutant L1T (L538T in MLV Env), all mutants displayed infectivity lower than wild-type MLV Env, demonstrating that even single conservative changes were enough to drastically impact Env function (Fig. 3C). Two mutants showed roughly half the infectivity of wild type, D8A (D545A in MLV Env) and G15N (G552N in MLV Env). As expected, a double glycine mutation had a more pronounced effect on infectivity than G15N alone. In our assays, the previously characterized double mutant, E14R + A20F (34, 36), resulted in significantly reduced infectivity (Fig. 3C).

### ISD mutants can still mediate cell-to-cell fusion

**Fig 4:**
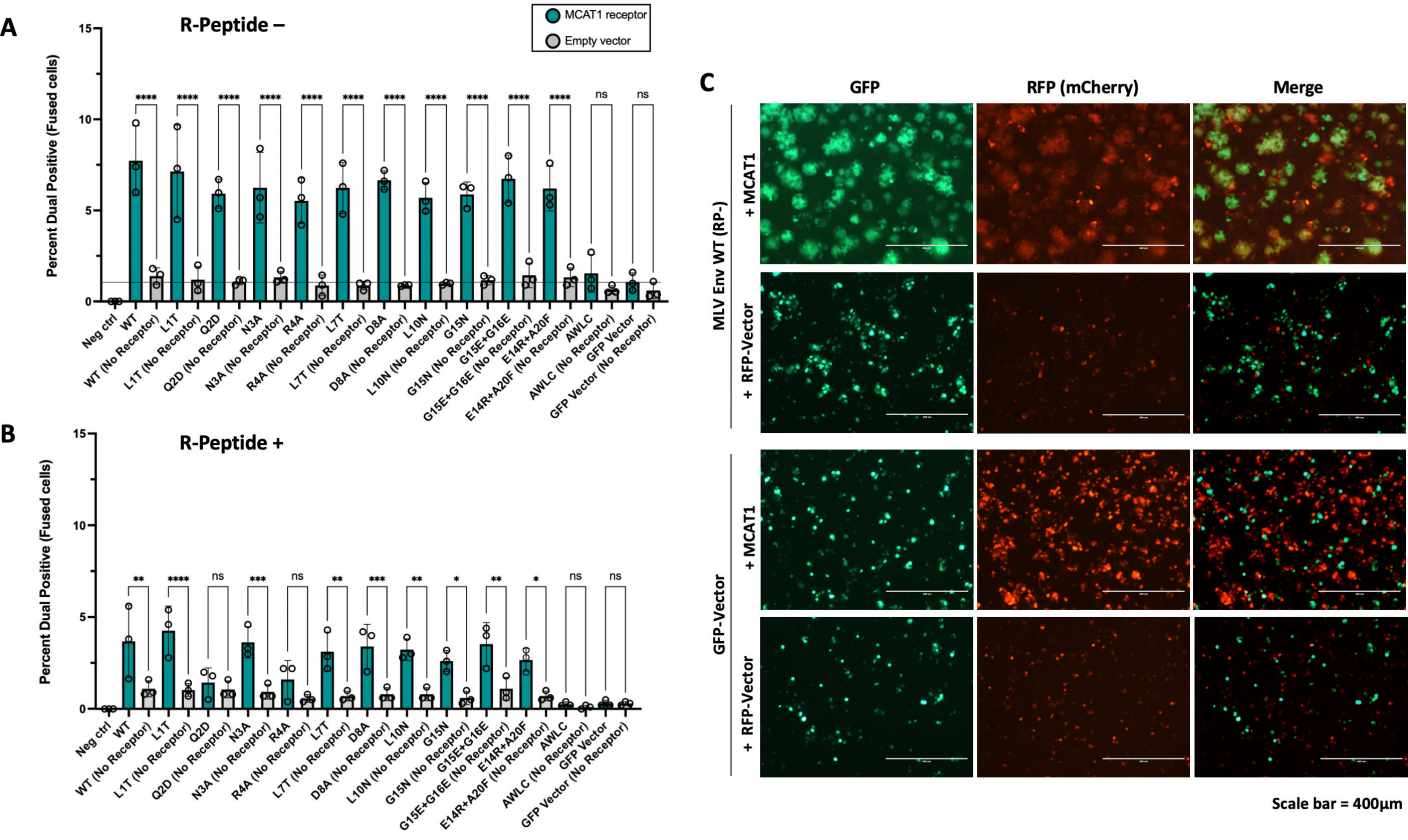
MLV ISD mutants are still fusogenic. (**A**) Cell-to-cell fusion ability of all Env mutants as measured via flow cytometry. To facilitate fusion, the R-peptide was removed from all Envs. HEK293T cells were transfected with either MLV Env in an IRES-GFP expression construct (ecoMLV-EnvAvi-pCGCG), the receptor MCAT1 in an IRES-mCherry expression construct (pMSCV-MCAT1-IRES-mCherry), or an empty mCherry vector (pMSCV-IRES-mCherry). Two days post-transfection, cell populations were resuspended and combined in flow cytometry tubes such that each Env was combined with MCAT1 and an empty vector. After 20 minutes, cells were pelleted and fixed in 2% paraformaldehyde, then sent for flow cytometry analysis. All events that were dual positive for both mCherry and GFP were considered recently fused cells. An empty GFP vector was used as a no-Env control. A line is drawn at this no-Env control for visual comparison. (**B**) Cell-to-cell fusion as performed in A but with the R-peptide intact. An ordinary one-way ANOVA with multiple comparisons was performed for A and B (**** =*P* < 0.0001, *** =*P* < 0.001, ** =*P* < 0.01, * =*P* < 0.05, ns = not significant). (**C**) Representative fluorescent images of syncytia formation between GFP and mCherry expressing cell populations, corresponding to wild-type MLV Env and a no Env control.

We next assessed the intrinsic fusogenicity of each mutant Env in a cell-to-cell fusion assay. In addition, an isomerization-deficient mutant was generated by substituting the first cysteine in the CWLC motif (CWLC to AWLC). This eliminates the free thiol of the motif and thus prevents isomerization of the intersubunit disulfide bond (16, 21). A C-terminal R-peptide corresponding to the last 16 residues of TM is normally cleaved by the viral protease during maturation. Because the R-peptide suppresses fusion prior to cleavage, the R-peptide of all Envs was removed via mutagenesis. All mutant Envs lacking an R-peptide showed fusion levels above negative controls and similar to wild type (Fig. 4A). Furthermore, fusion for each Env was also measured without the addition of the receptor MCAT1 into target cells. There was no evidence of receptor-independent fusion for any MLV Env mutant (Fig. 4). We further tested fusion for each mutant without removing the R-peptide and found that all mutants except Q2D and R4A showed basal levels of fusion above no-receptor controls (Fig. 4B). Taken together, these results indicate that mutations to the ISD did not reduce the intrinsic receptor-dependent fusogenicity of Env.

### ISD mutant Envs are incorporated into virions, but many have reduced levels of SU

**Fig 5:**
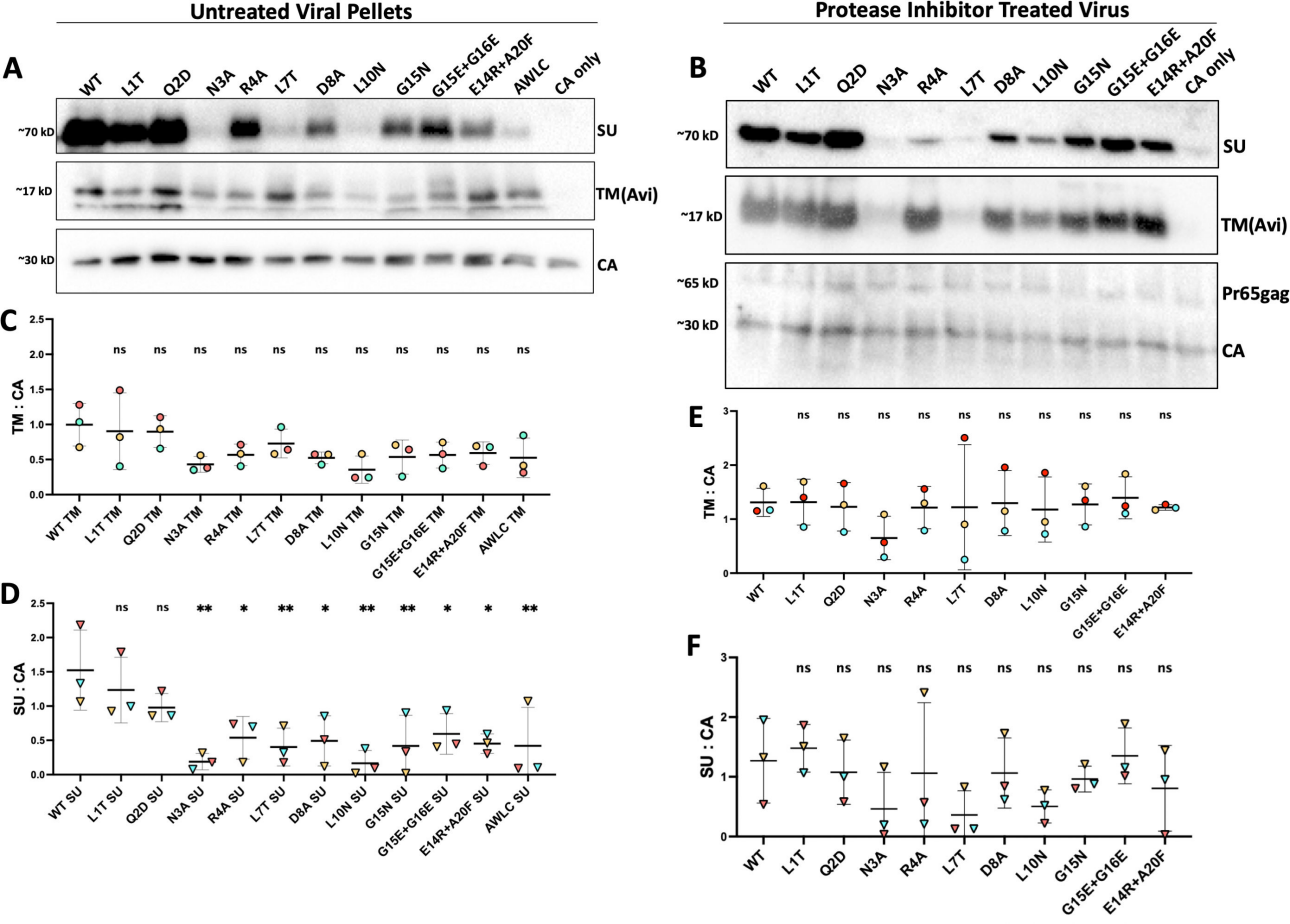
Incorporation of MLV Env ISD mutants into MLV particles. (**A**) Incorporation of Envelope mutants as seen on a western blot of viral pellets. Samples were treated with 2-mercaptoethanol to reduce all disulfide bonds for clarity in resolving TM. MLV SU was visualized with an anti-MLV-SU antibody, TM was visualized with the same anti-Avi stain used previously, and capsid was imaged using an anti-p30 antibody. (**B**) Western blot of viral pellets to measure incorporation of envelope mutants treated with a protease inhibitor, amprenavir, at 0.8 mM. MLV SU was visualized with an anti-MLV-SU antibody, TM was visualized with the same anti-Avi stain used previously, and capsid was imaged using an anti-p30 antibody. The blots in A and B are representative and correspond to the blue-colored data points in each corresponding graph, panels C, D, E, and F. (**C**) Ratio of TM to Capsid across three experiments (points colored by experiment). Densitometry was used to quantify levels of CA and TM for three sets of viral pellets. No significant difference was measured between wild-type and all mutant conditions. (**D**) Ratio of SU to capsid across three experiments (points colored by experiment). Densitometry was used to quantify levels of CA and SU for three sets of viral pellets. (**E**) Ratio of TM to capsid across three experiments treating virus with a protease inhibitor (points colored by experiment). Densitometry was used to quantify levels of CA and TM for three sets of viral pellets, using only the 30 kD capsid band for quantification. No significant difference was measured between wild-type and all mutant conditions using an ordinary one-way ANOVA with multiple comparisons. (**F**) Ratio of SU to capsid across the same three experiments as in E with points colored by experiment (** =*P* < 0.01, * =*P* < 0.05, ns = not significant. Ordinary one-way ANOVA with multiple comparisons).

We next investigated the levels of incorporation for each mutant by harvesting viral pellets from the infectious media of producer cells. These viral pellets were separated via SDS PAGE and visualized on a western blot stained with antibodies against SU, a C-terminal tag on TM, and capsid. All Envs were incorporated into virions as shown by the presence of TM (Fig. 5A). Compared to wild-type MLV Env, N3A and L10N have the lowest ratio of TM to CA; however, the ratio of TM to capsid for all mutants was not significantly different from wild type across multiple experiments (Fig. 5B). To determine if the cleavage of the R-peptide and attached Avi-tag had an impact on incorporation, we further measured ratios of TM and SU to capsid for virions produced in the presence of the viral protease inhibitor amprenavir at 0.8 mM (NIH-ARP). Amprenavir has previously been reported to block MLV protease from cleaving the R-peptide (37). When cleavage was blocked, no significant difference was seen between wild-type and mutant TM:CA ratios (Fig. 5B and C). Similarly, no significant difference was measured between wild-type and mutant SU:CA ratios. While we did not observe any major defects in TM incorporation in either untreated or protease-inhibited viral pellets, certain mutants had lower levels of SU present, particularly N3A, L7T, and L10N in untreated virions. In contrast, Q2D consistently displayed wild-type levels of SU (Fig. 5C). The reduced levels of SU suggest that the ISD mutations may destabilize the disulfide bond connecting the two subunits. This loss of SU is potentiated by the cleavage of the R-peptide.

### ISD mutations do not prevent the formation of the intersubunit disulfide bond

**Fig 6:**
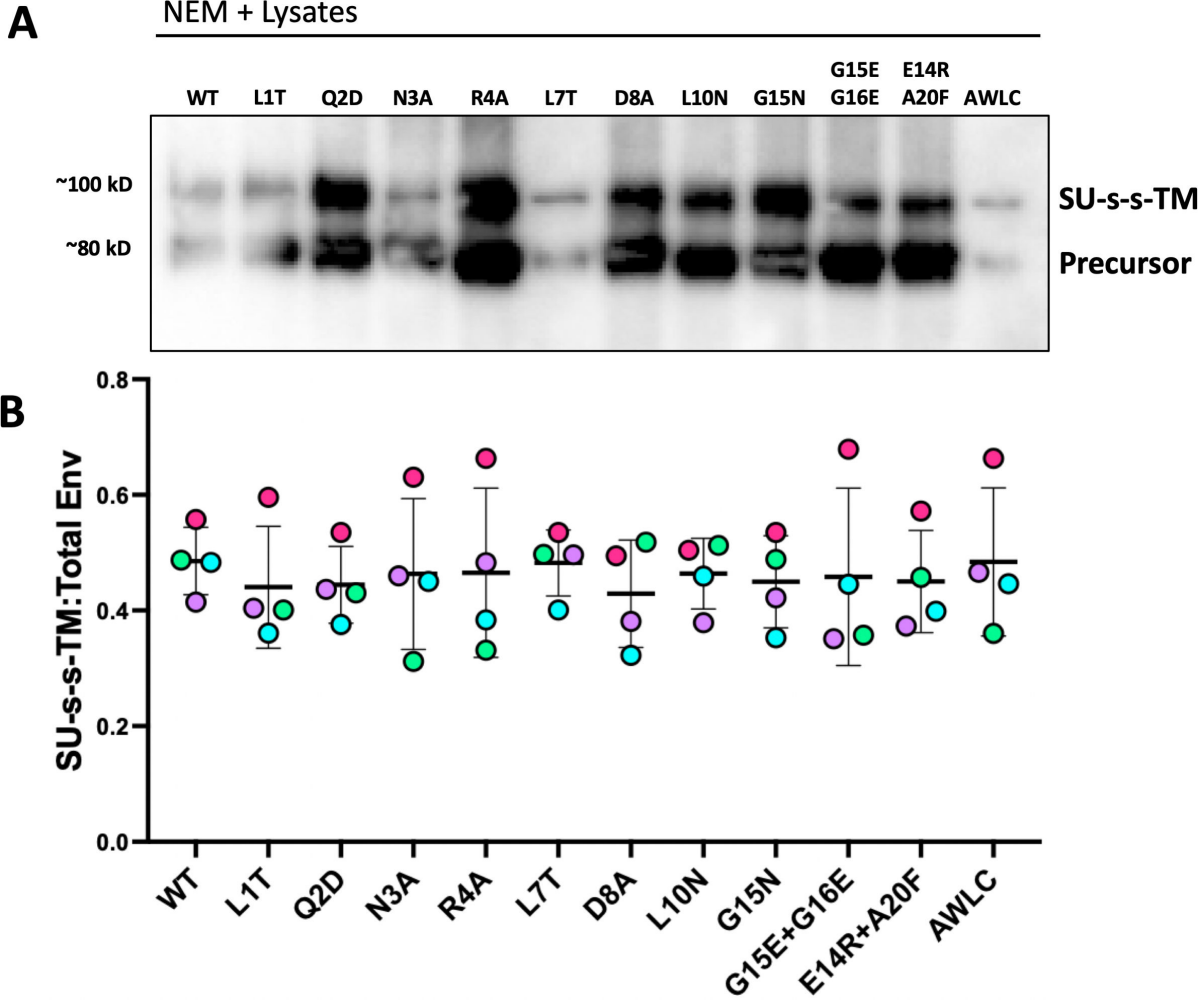
ISD mutations do not prevent the formation of the disulfide bond between SU and TM. (**A**) Representative blot of NEM-treated whole cell lysates of MLV ISD mutants, stained with anti-Avi to detect TM either in precursor or in disulfide-bonded Env. Disulfide-bonded Env migrates to around 100 kD, precursor protein to around 80 kD, SU alone would migrate to 70 kD but cannot be visualized with the Avi antibody. (**B**) The amount of disulfide-bonded Env for each mutant was quantified relative to the Env protein present in the lysate for four independent experiments (ns = not significant, one-way ANOVA with multiple comparisons).

To confirm the presence of disulfide bond between SU and TM, we ran all Env ISD mutants on a non-reducing SDS PAGE gel and used western blot to visualize Env. To confirm that the disulfide bond was forming for each mutant, Env-expressing cell lysates were treated with *N*-ethylmaleimide (NEM), an alkylating agent that prevents the reduction of disulfide bonds. As an additional control, we used the isomerization defective mutant (AWLC) to block dissociation of SU and TM. All samples were harvested in non-reducing conditions to preserve disulfide bonds, and blots were stained with an antibody against a tag on the C-terminus of TM which facilitates visualization of both the Env precursor and the disulfide bonded SU-TM heterodimer. To account for experimental variation in overall Env levels in these experiments, we determined the ratio of the disulfide-bonded form (SU-s-s-TM) to total Env (SU-s-s-TM + Precursor; Fig. 6).

A substantial amount of the disulfide intact form was present for wild type. Additionally, for all of the mutants, we observed the disulfide-bonded form in NEM-treated lysates (Fig. 6), indicating that the bond was still forming for these mutants.

### Mutations to the ISD promote premature dissociation of SU and TM

**Fig 7:**
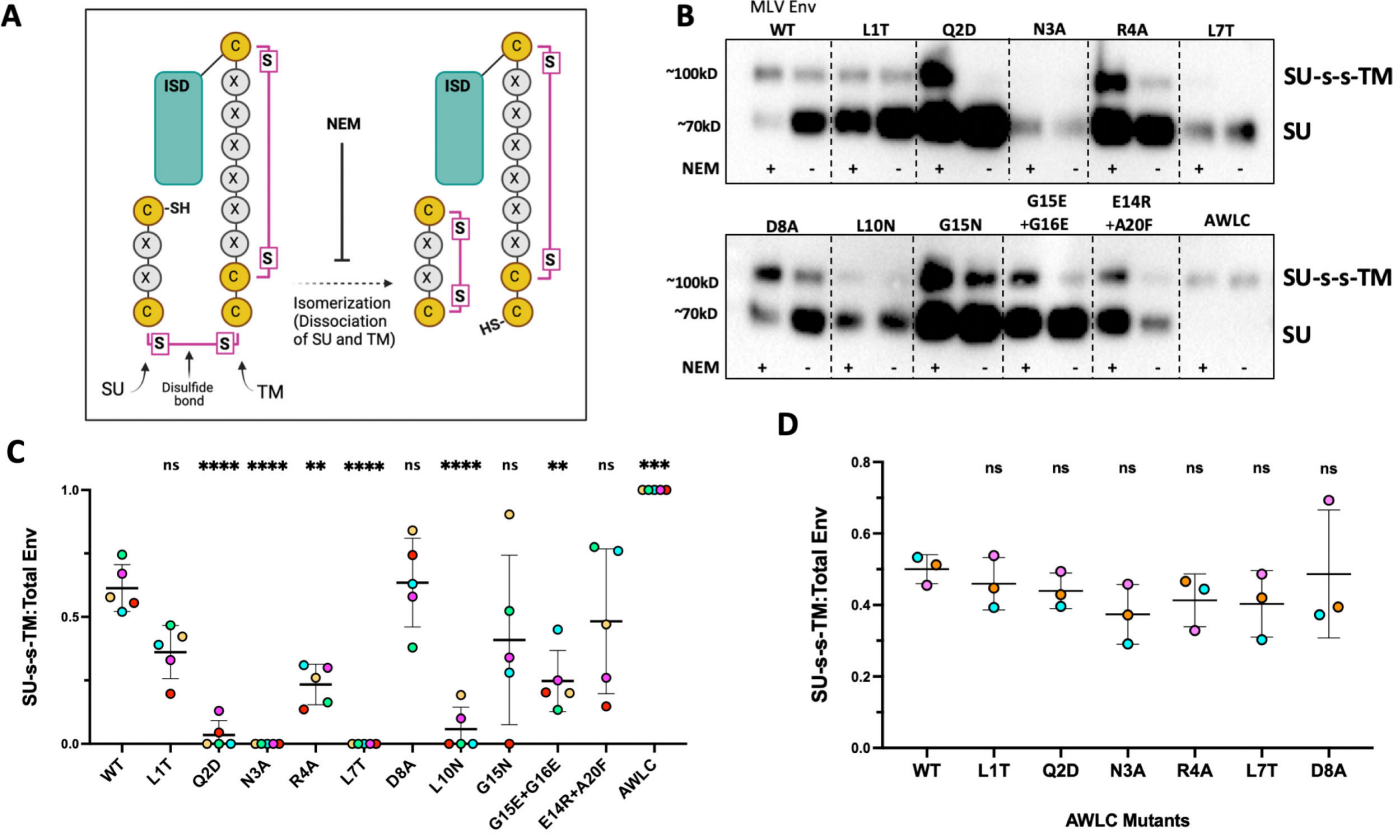
MLV ISD mutants have a less stable disulfide bond. (**A**) Schematic of conserved cysteine containing motifs and formation/isomerization of the disulfide bond between SU and TM subunits. (**B**) Non-reduced viral pellets were separated with SDS PAGE and visualized with western blot. NEM treatment was used to block isomerization of the disulfide bond, essentially fixing any bonded Env present in the samples. Non-treated samples were maintained in non-reducing conditions to preserve intact disulfide bonds. Mutant AWLC no longer has the free cysteine needed to isomerize and acts as a size control for the disulfide-bonded form of Env. Blots were stained with an anti MLV-SU antibody to enable visualization of disulfide-bonded Env and SU alone. (**C**) Densitometry of the disulfide bonded band compared to total protein present for untreated supernatant samples across five experiments (representative blot shown in panel **B**). Points are colored by individual experiments. (**D**) Densitometry quantification of three western blots of MLV Env ISD mutants combined with an AWLC mutation to block isomerization. Samples were harvested in non-reducing conditions and separated with SDS PAGE. Precursor and disulfide-bonded Env were detected with an anti-Avi antibody. Ratios of disulfide-bonded Env to precursor are shown for a selection of ISD mutants. Points are colored by individual experiements (ns = not significant, one-way ANOVA with multiple comparisons).

To determine the impact of ISD mutations on disulfide stability of Env incorporated into particles, we separated non-reduced viral pellets of all mutants with SDS PAGE and then used western blot to visualize Env with an intact disulfide bond, as well as SU after isomerization of the bond (Fig. 7A). These blots were stained with an MLV-SU antibody that can distinguish between SU alone and disulfide-bonded SU and TM (SU-s-s-TM; Fig. 7A). As with the lysates, a substantial amount of the disulfide intact form was visible for wild-type viral pellets, in both NEM and untreated conditions, and no dissociated SU was observed for the AWLC control (Fig. 7B).

In contrast, a majority of the mutants, particularly the ones incapable of mediating infection, demonstrated higher levels of SU than SU-s-s-TM. Additionally, these viral pellets allow for quantification of the ratio of disulfide-bonded Env to the total Env protein present as a measure of the general stability of the disulfide bond. When stability was quantified across four independent experiments for the untreated conditions, ISD mutants Q2D, N3A, R4A, L7T, and L10N all showed significantly lower levels of the disulfide-bonded form in viral pellets when compared to wild type (Fig. 7C).

To confirm that the loss of stability seen in our incorporation blots and non-reduced viral pellets was connected to isomerization of the disulfide bond, we combined several of the mutants that displayed the least amount of SU in incorporation results with the isomerization-resistant mutation AWLC. When combined with this mutation, the disulfide-bonded form of Env was more readily visualized on western blot. When the ratio of disulfide-bonded Env was quantified across multiple experiments, there was no longer a difference between wild-type and mutant Envs (Fig. 7D) demonstrating that the mutations to the ISD did not prevent the initial formation of the disulfide bond, but the loss of SU seen in Fig. 5 was due to early isomerization of the disulfide bond.

## DISCUSSION

The Env protein of MLV is the prototype for a large clade of retroviral entry proteins, which we refer to collectively as gamma-type Envs (6, 38). Gamma-type Envs are readily identified by the presence of (i) a metastable intersubunit disulfide bond and (ii) a highly conserved stretch of 26 residues in the TM subunit. These 26 residues comprise the putative immunosuppressive domain, or ISD (positions 1–17), and a CX_6_CC motif (positions 18–26). The combined ISD + CX_6_CC sequence is found in viruses of the *Alpharetrovirus*, *Gammaretrovirus*, and *Deltaretrovirus* genera. It is also found in a subset of viruses in the *Betaretrovirus* genus, including the type-D simian retroviruses (6, 15). Finally, this hallmark sequence is also found in the fusion subunit (GP2) of viruses in the *Filoviridae* family, including EBOV.

The three cysteines of the CX_6_CC motif are absolutely conserved, reflecting an essential role in the formation of two disulfide bonds. One is an intrasubunit bond, which forms between the first and second cysteines, and the other is a metastable, intersubunit bond that forms between the third cysteine and an unpaired cysteine in SU (or GP1, in the case of EBOV). In contrast, the function of the adjacent 17-residue ISD is not well defined. Deletions in the ISD region result in shedding of SU and complete loss of infectivity (35, 39). Consistent with those reports, we found that individual substitutions at highly conserved positions in the MLV ISD result in premature isomerization of the intersubunit disulfide bond. In most cases, the mutation also resulted in reduced levels of virion-associated SU and significant loss of infectivity. Specifically, we observed a correlation between instability of the SU-s-s-TM disulfide bond and loss of infectivity (*r* = 0.63, *P* = 0.03, Supp. Fig. 1C). Similarly, there was a correlation between shedding of SU and loss of infectivity (*r* = 0.71, *P* = 0.015, Supp. Fig. 1D). Based on these observations, we propose that the extraordinary conservation of the ISD reflects a fundamental role in regulating timing of isomerization of the intersubunit disulfide bond during entry and fusion by gammaretroviruses and other viruses with gamma-type Envs.

**Fig 8:**
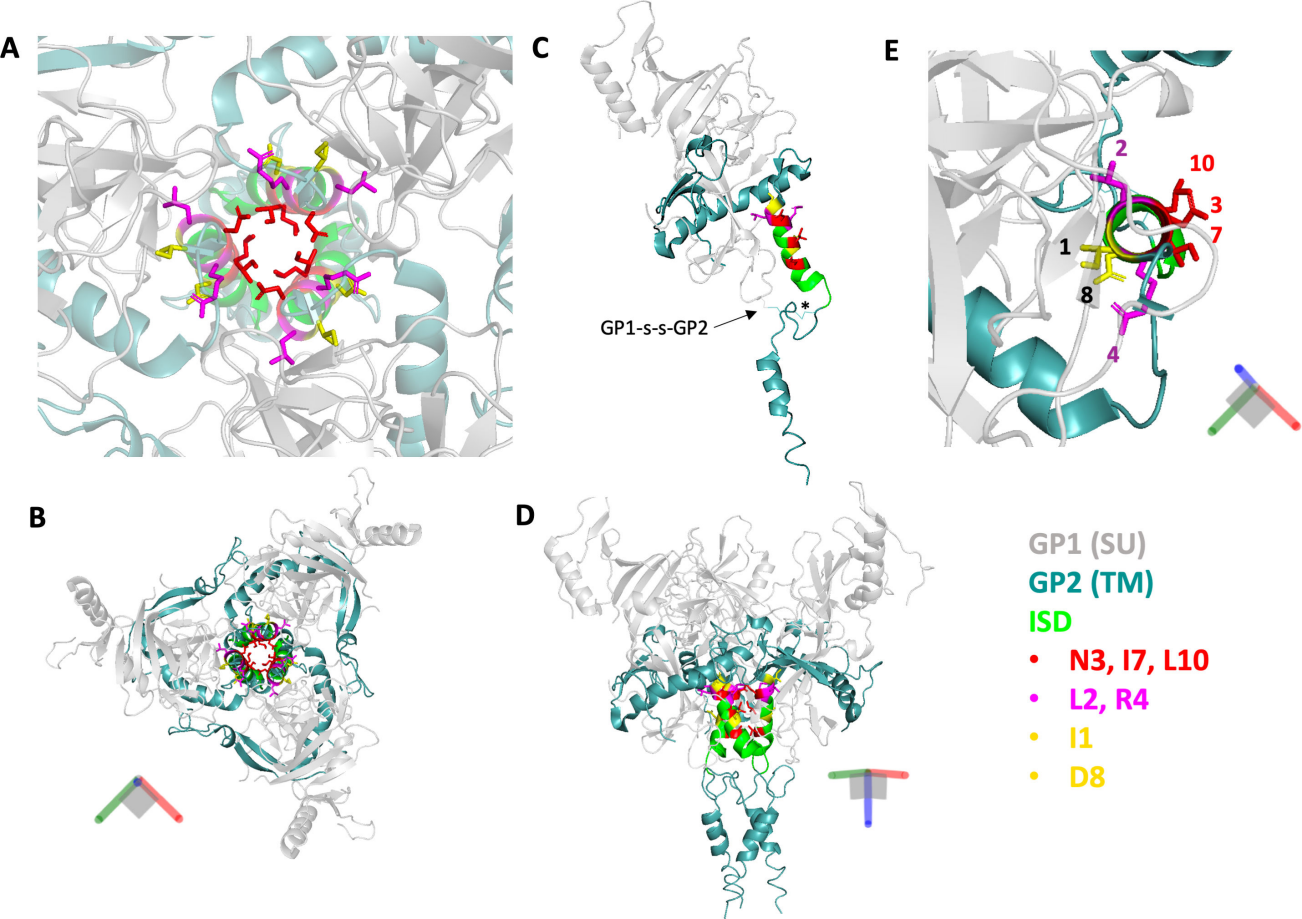
Structure of the pre-fusion Ebola GP trimer highlighting highly conserved residues of the ISD. (**A**) Top-down view of the central axis of the pre-fusion crystal structure of Ebola GP (6QD8). Side chains are shown for residues 1, 2, 3, 4, 7, 8, and 10 of the ISD. (**B**) Zoomed-out view of panel A. (**C**) Single heterodimer (GP1-s-s-GP2) with side chains shown as in A. The intersubunit bond is indicated with an arrow to label the linkage between GP1 and GP2, while the intrasubunit bond that forms between the first two cysteines of the CX_6_CC is called out by a star. (**D**) Zoomed-out view of panel C showing the full Ebola GP trimer. (**E**) Top-down view of the alpha helix formed by the ISD. Side chains are shown for residues 1, 2, 3, 4, 7, 8, and 10. For clarity, only one heterodimer is shown.

Published MLV Env structures currently lack the resolution to provide insights into the precise position of the ISD or its intermolecular contacts (37). However, a high-resolution pre-fusion structure of the EBOV GP trimer that includes the ISD sequence has been described (24). Side chains for amino acids occupying three of the highly conserved ISD residues, at positions 3, 7, and 10, are oriented toward the central axis of the trimer, whereas the side chains of residues at positions 1, 2, 4, and 8 are oriented toward different parts of the associated GP1 subunit (Fig. 8). Alpha fold models suggest that MLV Env may have a similar TM and ISD arrangement (Fig. S2). Notably, mutations in the MLV ISD at positions 3, 7, and 10 resulted in nearly identical phenotypes, including destabilization of the disulfide bond, the lowest SU:CA ratios, and loss of infectivity (Table 1). In contrast, the effect of mutations at MLV ISD positions 1, 2, 4, and 8 was variable, perhaps reflecting different interactions with SU. For example, L1T did not detectably destabilize the disulfide bond, did not have a significant effect on infectivity, and had an SU:CA ratio similar to wild type. D8A was similar to L1T but with a modest reduction in infectivity. Interestingly, Q2D destabilized the disulfide bond while retaining wild-type levels of virion-associated SU, and R4A also destabilized the disulfide bond, while retaining intermediate levels of virion-associated SU. Both Q2D and R4A displayed a reduced capacity for cell-cell fusion as compared to the other mutants when the R-peptide was intact, although this effect was not observed when the R-peptide was deleted. Finally, G15N had only a modest effect on infectivity, while the G15E/G16E double mutant resulted in a complete loss of infectivity. Comparing these observations suggests that different faces of the ISD helix play different roles; for example, based on our results and comparison to the EBOV structure, positions 3, 7, and 10 in the MLV ISD may allow interactions between the TM subunits along the trimeric axis, while some or all of the side chains at positions 1, 2, 4, and 8 may engage in non-covalent interactions between SU and TM.

**TABLE 1:** Summary of ISD mutant phenotypes compared to wild-type MLV Env^*a*^

Mutant	Processing	Incorporation (TM:CA)	Disulfide stability	Fusion	Infectivity
L1T	++	++	++	++	++
Q2D	++	++	−	+	−
N3A	+	+	−	++	−
R4A	++	++	−	+	−
L7T	+	+	−	++	−
D8A	++	++	++	++	+
L10N	++	+	−	++	−
G15N	++	++	++	++	+
G15E and G16E	++	++	−	++	−
E14R and A20F	++	++	++	++	−
AWLC	++	++	+++	−	−
Wild type	++	++	++	++	++

^*a*^ ++ is not significantly different from wild type, – is significantly different from wild type (*P* < 0.05), and + is lower than wild type but significantly above negative controls. AWLC is hyper-stable (unable to isomerize) and therefore listed as +++.

As with influenza A virus HA, the prototypical class 1 fusogen, binding of MLV Env to its receptor initiates unfolding of TM and insertion of the fusion peptide into the host cell membrane (6). However, in contrast to IAV HA, refolding of MLV TM into the post-fusion structure requires isomerization of the SU-s-s-TM disulfide bond, and mutations that “trap” the extended conformation of MLV TM can be rescued by treatment with a reducing agent or by exposure to a soluble receptor-binding domain provided *in trans* (23, 40, 41). This is distinct from IAV, where the disulfide linking the HA1 and HA2 subunits remains intact throughout the fusion and entry process (42). Moreover, IAV HA2 does not contain the characteristic ISD and CX_6_CC motifs that define the gamma-type clade of entry proteins. Therefore, the SU-s-s-TM bond in gamma-type entry proteins is functionally distinct from the IAV HA1-s-s-HA2 disulfide bond. In the case of IAV, the bond maintains a stable covalent association between the receptor-binding and fusion subunits throughout fusion and entry, whereas in MLV, the bond is metastable and reduction of the bond serves as a critical “switch” regulating timing of a late step in the fusion and entry process (20, 43).

During MLV fusion and entry, the reduction of the intersubunit disulfide is coupled to the oxidation of the CWLC motif (16, 18, 20). Pinter et al. previously noted that the CWLC in MLV SU is analogous to the CXXC active sites of cellular protein disulfide isomerases (PDIs), which also become oxidized during the reduction of substrate disulfide (18). Indeed, the intersubunit bond that forms between the CX_6_CC in TM and the CWLC in SU resembles the mixed disulfide intermediate that forms between a PDI active site CXXC and a protein substrate thiol undergoing a coupled reduction/oxidation reaction (18, 44, 45). Our results indicate that the destabilizing effect of mutations in the ISD motif of TM requires the intrinsic isomerization activity provided by the CWLC motif in SU. Therefore, we propose that the ISD functions to prevent premature reduction of the intersubunit disulfide bond by the CWLC. In this model, the cascade of structural rearrangements initiated when SU binds a receptor may serve to relieve ISD-mediated suppression of isomerization, which in turn displaces SU and allows refolding of TM into the post-fusion conformation. Based on extrapolation from the prefusion EBOV trimer structure, we speculate that this involves dynamic interactions between SU and the ISD helix involving residues at positions 2, 4, and/or 8, and between the three ISD helices involving residues at positions 3, 7, and 10. The former could also be involved in responding to the receptor-induced changes in SU that lead to the initial extension of TM and insertion of the fusion peptide. Comparison of pre- and post-fusion EBOV GP structures suggests that the glycines at positions 15 and 16 in the MLV ISD provide structural flexibility necessary for both the initial extension of TM and, upon isomerization of the bond, its subsequent refolding into the post-fusion, six-helix bundle. Additional high-resolution structural data, particularly structures of the disulfide-bonded, “TM-extended” intermediate, would greatly improve our understanding of the ISD and its contributions to the gamma-type entry mechanism.

Alpharetroviruses (e.g., avian leukemia virus, ALV) and filoviruses (e.g., EBOV) represent a variation on the gamma-type Env (6, 38). These viruses have the labile disulfide bond and the ISD+CX_6_CC motif, but both lack the CWLC isomerization motif found in MLV. One intriguing possibility is that ALV and EBOV depend on cellular PDIs expressed in the endocytosis pathway to reduce the intersubunit disulfide bond. Notably, work on both viruses suggests the existence of an unidentified host factor required for the final step in fusion (20, 46–48). Based on our characterization of the MLV ISD, we propose that the ISDs of ALV TM and EBOV GP2 also function to stabilize the intersubunit disulfide bonds in these viruses, and receptor binding (perhaps in combination with a drop in pH) repositions the ISD such that host-encoded PDIs can then access and reduce the disulfide.

As Env is, in a sense, “spring-loaded” for fusion, the control of fusion, and protection of the disulfide bond during Env trafficking, and virion assembly may be necessary to prevent premature dissociation of the Env subunits. Multiple mechanisms may exist in Env as controls to ensure fusion occurs only in specific contexts. During viral maturation, the MLV protease removes a short peptide from the C-terminal cytoplasmic tail of TM (the R-peptide). R-peptide cleavage results in a significant increase in fusion capability (37, 49, 50). This effect was recapitulated by experimentally deleting the R-peptide from MLV Env ISD mutants for use in cell-cell fusion assays. Interestingly, inhibiting cleavage of the R-peptide with amprenavir may increase the stability of ISD mutants. Taken together, these observations confirm that the R-peptide operates as a dominant control over fusion, and this control involves the disulfide bond (50). We also observed increased shedding of SU from virions in the absence of amprenavir, suggesting that cleavage of the R-peptide may potentiate destabilization in ISD mutants; this also raises the possibility that cleavage of the R-peptide from wild-type Env may be necessary from normal, receptor-induced isomerization of the disulfide during fusion and entry. Indeed, cleavage of the R-peptide causes a significant rearrangement of the TM ectodomains in MLV Env trimers, which is likely to include repositioning of the three ISDs and their respective side chains (37).

Interestingly, when combined with an R-peptide deletions, the ISD mutant Envs demonstrated levels of receptor-dependent cell-cell fusion similar to wild-type Env. Possibly, differences between cell-cell fusion and virion-mediate fusion compensate for, or obscure, the effects of the ISD mutations. At a minimum, the results of the cell-cell fusion assay indicate that the ISD mutations did not grossly impact the intrinsic fusogenicity of TM. This is consistent with a model, wherein the presence of the disulfide bond normally blocks a step in entry that precedes membrane fusion.

What about immunosuppression? The first reports describing the putative immunosuppressive activity of a synthetic peptide corresponding to the MLV ISD also noted the conservation of the sequence in other retroviruses (25). Here, we show that this conservation extends beyond mammalian retroviruses, as evidenced by its presence in ERV loci found in the genomes of birds, amphibians, and fish. The ISD motif is also ancient—the sequence is found in human Syncytin-1 and Syncytin-2, which are >25 million and >40 million years old, respectively; it is also found in HEMO, another ERV-encoded Env protein that is >100 million years old, and in the *percomorf* locus of ray-finned fish, which is also >100 million years old (51–54). Genes of the innate and adaptive immune systems are among the most divergent loci when compared across vertebrate lineages (55). Thus, it is challenging to conceive of an aspect of immunity that would be sufficiently conserved in birds, fish, amphibians, and mammals to be targeted by a single, highly conserved motif such as the ISD. The antiquity of the ISD sequence is also at odds with the evolutionary “arms-race” pattern typical of the evolution of proteins involved in molecular-level virus-host interactions (55). Often, viral factors that interact with the host immune system must continuously adapt to keep up with evasive counter-adaptations in the relevant host factors. The extreme conservation of the ISD and the dramatic effect of mutating specific residues in the ISD on viral infectivity are more consistent with highly conserved, fundamental contribution to viral infection and replication. However, it is worth noting that the immunosuppressive effects reported thus far have involved a limited number of mammalian retroviruses and mammalian cells (or murine models); therefore, it is possible that the ISDs of these viruses also provide an immunosuppressive function in addition to a fundamental role in regulating fusion and entry. However, the latter is still a more compelling explanation for the observed conservation of the ISD sequence and its invariant association with the intersubunit disulfide bond across hundreds of millions of years of divergent viral evolution. Given the ubiquitous nature of gamma-type Envs, a deeper understanding of the nature and function(s) of the ISD will greatly enhance our understanding of the large clade of class I fusogens.

## MATERIALS AND METHODS

### Plasmids

Moloney MLV Env was taken from pCL-Eco (Addgene) and placed in a pCGCG backbone upstream of an IRES and GFP via homologous recombination (using New England Biolab’s HiFi Assembly kit) to create ecoMLV-EnvAvi-pCGCG. An Avi epitope tag was appended to the C-terminal end of Env after the R-peptide. All subsequent mutants were generated from this plasmid. For infections, pCIG3N (N-tropic MLV gag-pol from Addgene) and pLXIN-GFP were used with Env to produce virus. MCAT1 in an IRES-mCherry expression construct for fusion assays was created via homologous recombination of MCAT1 from ppMCAT1 (Addgene) and pMSCV-IRES-mCherry (Addgene) to create pMSCV-MCAT1-IRES-mCherry.

### Mutagenesis

The Q5 Site Directed Mutagenesis kit from New England Biolabs was used to generate all single and double amino acid mutants. The NEBase changer online tool (https://nebasechanger.neb.com/) was used for mutagenic primer design, and all primers were generated through Integrated DNA Technologies. All plasmids were grown in TOP10 competent cells, and all mutants were verified via forward and reverse sequencing performed by Eton Biosciences.

### Cell culture

HEK293T cells and NIH 3T3 cells were cultured and maintained using Dulbecco’s Modified Eagle Medium with 10% fetal bovine serum and 1% Penicillin/Streptomycin and 1% added L-Glutamine. Cells were grown at 37°C. All transfections used Genjet (SignaGen) at a 1:3 ratio of DNA to Genjet reagent and followed the SignaGen Genjet transfection protocol optimized for HEK293T cells. For treatment of producer cells with a viral protease inhibitor, amprenavir (NIH AIDS Reagent Program) was added to D10 to a total concentration of 0.8 mM and was used as growth medium for producing virus. Viral pellets were then harvested as described below.

### Infections and flow cytometry

Producer cells were seeded for 60%–70% confluence 24 prior to transfection. Transfections followed the SignaGen Genjet transfection protocol. Env (ecoMLV-EnvAvi-pCGCG), MLV gag-pol (pCIG3-N), and a packageable GFP reporter (pLXIN-GFP) were transfected into HEK293T cells at a ratio of 1:2:2, respectively. The transfection mix was left on cells overnight and replaced with fresh D10 18 hours post-transfection. After 24 and 48 hours, producer cells were checked for GFP fluorescence to ensure proper transfection. Target cells were seeded at roughly 50,000 cells per well into 12-well plates, 24 hours prior to infection. After 72 hours post-transfection, infectious media was collected from the producer cells and centrifuged for 5 minutes at 1,500 rpm to remove cells and debris. Media was then aspirated off target cells, and 500 µL of infectious media was added to each well. This media was then diluted with 500 µL of fresh D10 24 hours later. Target cells were harvested for flow cytometry 72 hours post-infection. To harvest cells, each well was trypsinized, resuspended in D10, and pelleted in FACS tubes before being resuspended in 150 µL of 4% paraformaldehyde. Each sample was run on a BD FACSAria flow cytometer and subsequently gated for living cells, single cells, and GFP-expressing cells using FlowJo v8.7.3 BD (Biosciences). An uninfected control was used to establish all gates which were then applied to all samples. Tests for normality and an ordinary one-way ANOVA with all conditions compared to wild type were performed using GraphPad Prism software (v.10.0.2).

### Cell-cell fusion assay

HEK293T cells were transfected with an Env (ecoMLV-EnvAvi-pCGCG). HEK293T cells do not have the MCAT1 receptor used by ecotropic MLV. After 18 hours, these cells were resuspended and combined with HEK293T cells transfected with the MCAT1 receptor (pMSCV-MCAT1-IRES-mCherry) at even ratios of Env to receptor-expressing cells. The mixed population cell suspension was allowed to incubate in FACS tubes for 20 minutes before being pelleted and fixed in 2% paraformaldehyde. The percent of GFP positive (Env), mCherry positive (MCAT1), and dual positive (Env and MCAT1) cells in each sample was measured using flow cytometry. Dual positive events were considered fused cells. As a control for receptor independent fusion, each Env was also combined with HEK293T cells transfected with an empty mCherry vector (pMSCV-IRES-mCherry). An ordinary one-way ANOVA was performed with multiple comparisons using GraphPad Prism software (v.10.0.2). Additionally, combined cell populations were imaged in six-well plates using an EVOS M5000 imaging system at 10× magnification.

### Western blots

For initial test expressions of Env proteins, HEK293T cells were transfected with Env-expressing plasmids using the transfection protocol detailed above. Whole cell lysates were harvested 72 hours post-transfection and lysed directly in 2× Laemmli buffer containing 2-Mercaptoethanol. For non-reduced viral pellets, transfection of producers was performed as detailed above. Three days after transfection, media was collected from the producer cells, filtered to remove cellular debris, and centrifuged at 14,000 rpm (20,000 × *g*) and 4°C for 3 hours to pellet virions. Viral pellets were then lysed using IP lysis buffer and eluted into 2× Laemmli buffer without 2-Mercaptoethanol or any other reducing agent. All samples were run on 10% polyacrylamide gels at 90V for 2.5 hours in Tris Glycine SDS buffer. Proteins were transferred to a polyvinylidene fluoride (PVDF) membrane using 100V for 1.5 hours. Membranes were blocked using 5% milk in 1× phosphate buffered saline and subsequently incubated in primary antibody solution overnight at 4°C. Primary antibody was washed off using 1× PBS with Tween at 0.05%, and blots were incubated in horseradish peroxidase (HRP) conjugated secondary antibody solution for 1 hour at room temperature. An Enhanced Chemiluminescence kit (Cytiva) was used to image all blots. BioRad Image Lab software (v6.1) was used to perform densitometry for band quantification, and all statistical analyses were done using GraphPad Prism software (v.10.0.2) using an ordinary one-way ANOVA comparing all conditions to wild type.

### Antibodies

All antibody dilutions were made with 5% milk-PBS. A rabbit IgG anti-Avi polyclonal antibody from Genscript was used at a dilution of 1:3,000 in combination with a goat anti-Rabbit IgG-HRP monoclonal antibody at 1:2,000. To visualize MLV SU, a goat anti-MLV SU monoclonal (80 S-019, Gift from Dr. Monica Roth) was used at 1:2,000 in combination with a donkey anti-Goat IgG-HRP secondary antibody at 1:2,000. Capsid was visualized with a rat anti-MLV p30 (gag) from Absolute Antibody and an anti-Rat IgG HRP from Cell Signaling Technologies. A mouse IgG anti-β-actin from Invitrogen was used at 1:3,000 in combination with a goat anti-Mouse IgG-HRP conjugated secondary at 1:2,000.

### Bioinformatics

All sequences were found on NCBI. Protein alignments and phylogenies were run using Geneious Prime 2022.2.2 (https://www.geneious.com). Accession numbers in order of alignment are as follows:

Ebola virus - Q05320, ALV - K7SHS6, RSV - BAD98245, Avian ERV - CAB58112.1, Fur seal ERV - XP_025743503.1, Clawed Frog ERV - QXP50143.1, Clingfish ERV - XP_028321565.1, Stickleback fish ERV - XP_040024727.1, Zebrafish ERV - AF503912, Toad ERV - XP_040289417.1, Rainbowfish ERV - XP_041856078.1, Marmoset ERV - ACI62863.1, Gorilla ERV - AGI61275.1, Colobus monkey ERV - NP_001295060.1, Dog ERV - XP_022270294.1, Bonobo syncytin-1 (ERV) - NP_001291475.1, Human syncytin-1 - Q9UQF0.1, Simian retrovirus 3 (MPMV) - NC_001550.1, RD114 retrovirus - ABS71857.1, Avian spleen necrosis virus - P31796.1, REV - QXV86750.1, Bat gammaretrovirus - AHA85401.1, Squirrel monkey retrovirus - NP_041262.1, Human syncytin-2 - P60508.1, HTLV-1 - Q03816.1, HTLV-2 - AAL30506.1, FeLV - AYG96595.1, Endogenous murine leukemia virus (AKR) - AAB03092.1, Moloney MLV - NC_001501.1, Friend MLV - P26804.1, Mink cell focus-forming virus - AAO37271.2, Xenotropic MLV - AEI59727.1, Porcine endogenous retrovirus - ACD35952.1, Boar ERV - AAQ88184, GALV - ALV83307.1, KoRV - ALX81658.1, and Marmot ERV XP_015345157.1.

Structure predictions were created using Alphafold2 run through ColabFold (https://colab.research.google.com/github/sokrypton/ColabFold/blob/main/AlphaFold2.ipynb#scrollTo=kOblAo-xetgx), using the MoMLV Env sequence with a six-residue linker (GAGAGA) between SU and TM after the furin cleavage site. Predicted and crystal structures were graphically analyzed with PyMol (v2.4.2 Schrödinger, Inc.) software. The Ebola GP crystal structure 6QD8 was obtained from RCSB Protein data bank. This crystal structure includes bound antibody fragments which were omitted from our analyses for clarity but may have an impact on the structure of Ebola GP.

## Data Availability

All data generated as part of this study are available from the corresponding author upon request.

## SUPPLEMENTAL MATERIAL

Supplemental figures jvi.00989-24-s0001.docx Figures S1 to S3.

ASM does not own the copyrights to Supplemental Material that may be linked to, or accessed through, an article. The authors have granted ASM a non-exclusive, world-wide license to publish the Supplemental Material files. Please contact the corresponding author directly for reuse.
