# Supplementary material for: Conserved residues of the immunosuppressive domain of MLV are essential for regulating the fusion-critical SU-TM disulfide bond
Source: J Virol. 2024 Oct 29;98(11):e00989-24. doi: 10.1128/jvi.00989-24 (PMC11575397; doi:10.1128/jvi.00989-24)
Supplement: Supplemental figures — Figures S1 to S3. [file jvi.00989-24-s0001.docx]

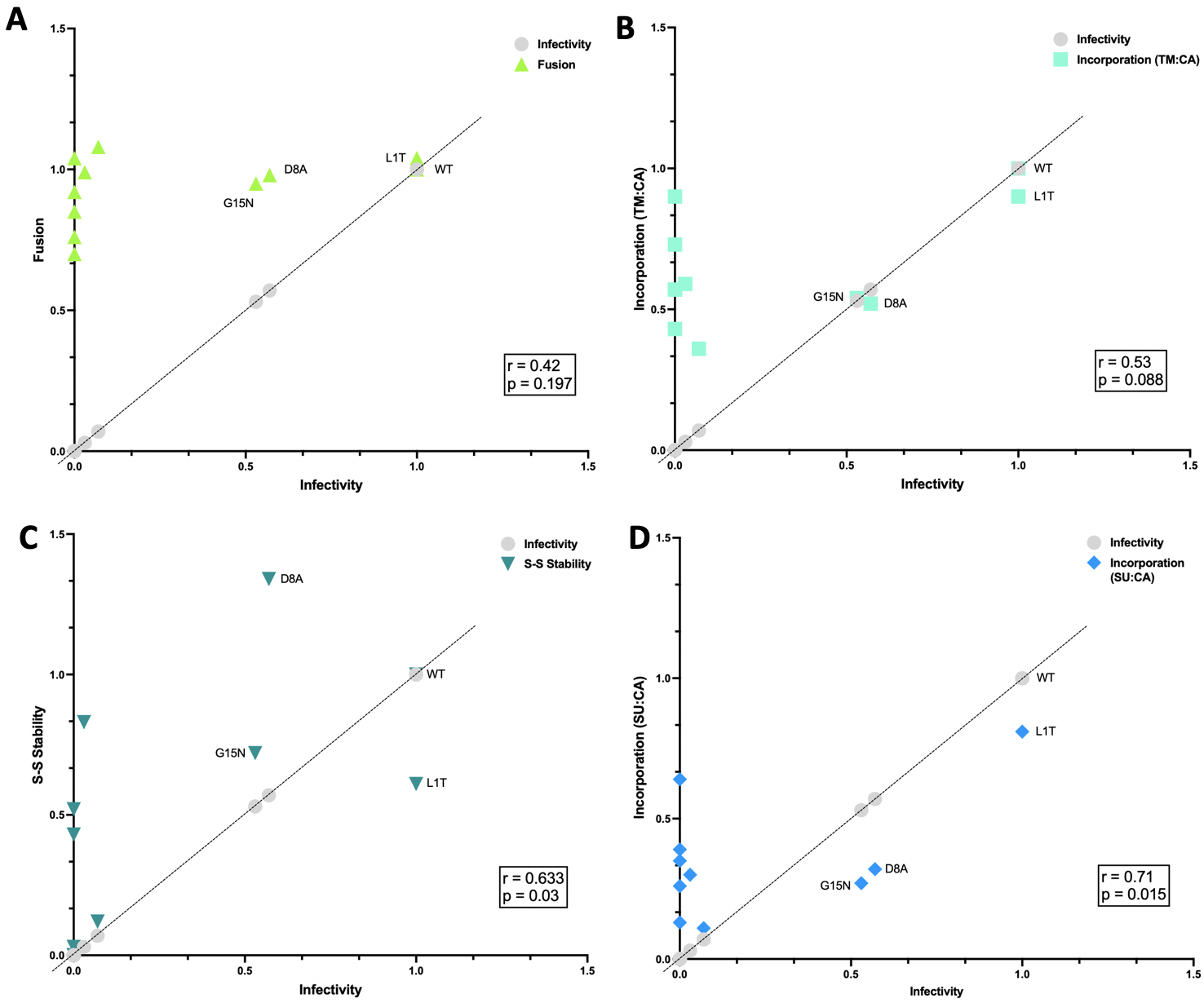


**Supplemental Figure 1: Correlation of ISD mutant phenotypes with infectivity**

A) XY data of the correlation between fusion data (Figure 4) and infectivity data (Figure 3c). B) XY data of the correlation between TM:CA data (Figure 5c) and infectivity data (Figure 3c). C) XY data of the correlation between disulfide stability data (Figure 7c) and infectivity data (Figure 3c). D) XY data of the correlation between SU:CA data (Figure 5d) and infectivity data (Figure 3c). All data sets were normalized to wild type.


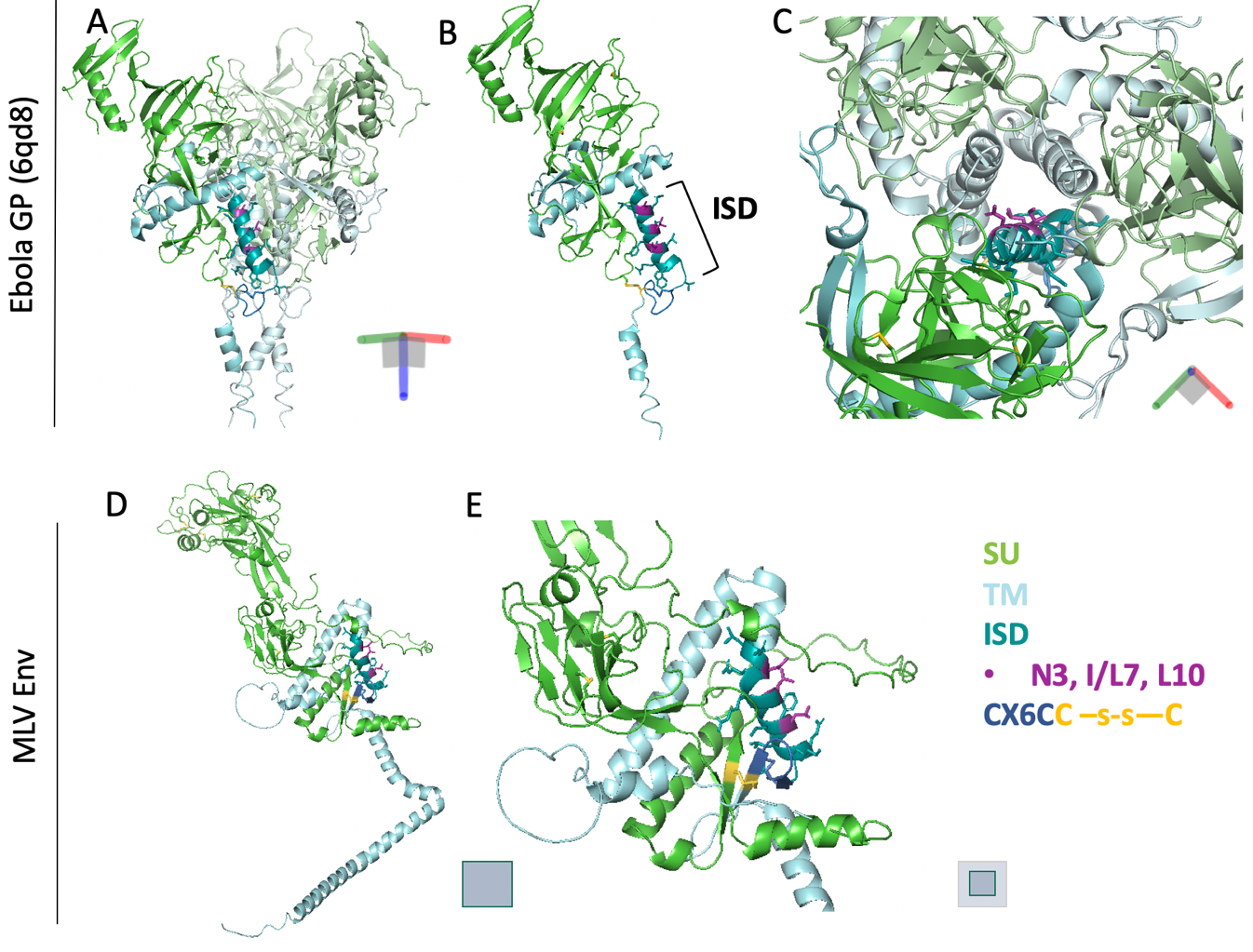


**Supplemental Figure 2: Pre-fusion structures of Ebola GP and Alphafold predictions of MLV Env show conserved structure and position of the ISD**

A) A single pre-fusion heterodimer of Ebola GP1 and GP2 (6qd8) colored by subunit. Residues N3, I7 and L10 of the ISD are highlighted in purple. B) Pre-fusion trimer of Ebola GP (6qd8) colored by subunit. C) Top-down view of the same structure from panel B, with side-chains visible for the residues of the ISD. Residues N3, I7 and L10 of the ISD are highlighted in purple. D) Alphafold prediction of a “pre-fusion” heterodimer of MLV Env, colored by subunit. E) Zoomed in view of the same structure from panel D to display ISD sidechains. Residues N3, L7 and L10 of the ISD are highlighted in purple.


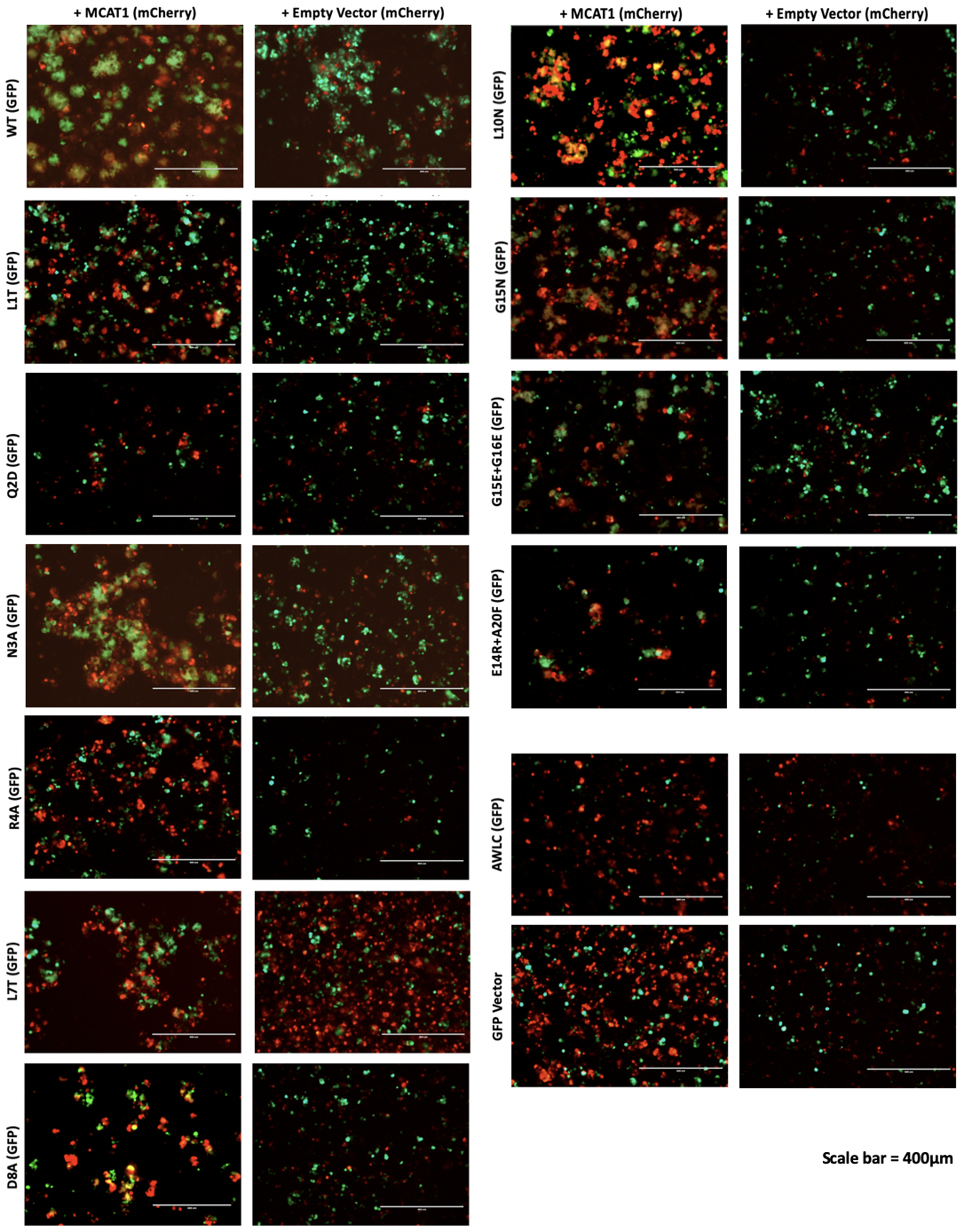


**Supplemental Figure 3: Two-color imaging shows MLV ISD mutants are still capable of fusion when the R-peptide is removed**

Merged fluorescent images of syncytia formation between GFP and mCherry expressing cell populations taken with an EVOS M5000 imaging system with a 10X objective. Env plasmids express GFP from an IRES while MCAT1 plasmids express mCherry from an IRES. An empty mCherry vector was used as a no-receptor control. All scale bars are equivalent to 400µM
